# Quantitative Evaluation of Ultrasound-Assisted Extraction of 1,3-β-glucans from *Dictyophora indusiata* Using an Improved Fluorometric Assay

**DOI:** 10.3390/polym11050864

**Published:** 2019-05-13

**Authors:** Yuan Fu, Shang Lin, Min Lu, Si-Yu Wei, Jia Zhou, Li Zhao, Qing Zhang, De-Rong Lin, Yun-Tao Liu, Hong Chen, Wen Qin, Ding-Tao Wu

**Affiliations:** Institute of Food Processing and Safety, College of Food Science, Sichuan Agricultural University, Ya’an 625014, Sichuan, China; yuanffuy@163.com (Y.F.); slsicau@163.com (S.L.); mlsicau@163.com (M.L.); SY_Weisicau@163.com (S.-Y.W.); jzsicau@163.com (J.Z.); zhaoli0608@126.com (L.Z.); zhangqing@sicau.edu.cn (Q.Z.); lindr2018@sicau.edu.cn (D.-R.L.); lyt_taotao@163.com (Y.-T.L.); chenhong945@sicau.edu.cn (H.C.); qinwen@sicau.edu.cn (W.Q.)

**Keywords:** *Dictyophora indusiata*, 1,3-β-glucans, ultrasound-assisted extraction, fluorometric assay, aniline blue

## Abstract

In the present study, an improved fluorometric assay based on aniline blue dye for the specific, accurate, and rapid quantification of 1,3-β-glucans in edible mushrooms was developed and fully validated. Furthermore, the improved method was successfully applied for the quantitative evaluation of water soluble 1,3-β-glucans extracted from *Dictyophora indusiata* by ultrasound-assisted extraction (UAE) with response surface methodology. Results showed that the improved method exhibited high specificity, accuracy, precision, repeatability, and stability, as well as a wide calibration range of 10–600 µg/mL (*R*^2^ > 99.9%). The maximum extraction yields of water soluble 1,3-β-glucans (1.20%) and total polysaccharides (5.41%) were achieved at the optimized extraction parameters as follows: ultrasound amplitude (56%), ultrasound extraction time (15 min), and ratio of liquid to raw material (22 mL/g). The results suggest that the improved fluorometric assay has great potential to be used as a routine method for the quantitative evaluation of 1,3-β-glucans in edible mushrooms and that the UAE method is effective for the extraction of 1,3-β-glucans from edible mushrooms.

## 1. Introduction

*Dictyophora indusiata*, a saprophytic fungus that belongs to the Phallaceae, is an edible mushroom widely used in China and other Asian countries [1]. Due to its attractive appearance and high nutritional value, *D. indusiata* is also regarded as “queen of the mushrooms” [2]. It has also received considerable attention in the biopharmaceutical area because of its medicinal effects, such as mental tranquilization, antitumor, anticorrosive, and enhanced immunity effects [3]. Generally, polysaccharides, which possess various pharmacological properties, are considered the main bioactive ingredients in *D. indusiata* [4]. Indeed, the branched 1,3-β-glucan is considered the most important polysaccharide in *D. indusiata*, due to its distinctive antitumor, immunomodulatory, and antioxidant activities [1,5,6,7,8]. The health benefits of branched 1,3-β-glucans from *D. indusiata* have attracted much attention [4]. Therefore, it is necessary to develop efficient methods for their extraction and quantification, which is helpful for the improvement of their performance in the functional/health foods area and beneficial for their quality control.

Traditional hot water extraction (HWE) is a popular method for the extraction of polysaccharides and has been applied for the optimization of total polysaccharide extraction from *D. indusiata* [9]. However, HWE is usually associated with a higher temperature, longer extraction time, and lower extraction efficiency [10]. Recently, ultrasound-assisted extraction (UAE) has been considered to be a powerful technique for the extraction of bioactive polysaccharides from natural resources [10,11,12,13]. Due to the cavitation in a strong acoustic field, UAE has a lot of advantages, such as increasing the diffusion, having a short extraction time, and being efficient and environmentally friendly [14]. Generally, the extraction efficiency of UAE is influenced by various factors, such as the ultrasound amplitude, the ultrasound extraction time, and the ratio of liquid to raw material, and their effects may be either independent or interactive [12]. Response surface methodology (RSM) has been described as an effective statistical technique for the optimization of the polysaccharide extraction process when the independent variables have a combined effect on the desired response [15,16]. Therefore, UAE with RSM could be a good approach for the extraction of water soluble 1,3-β-glucans from *D. indusiata*.

The well-known phenol sulfuric acid assay has been widely applied for the quantification of polysaccharides from edible mushrooms. However, this method has poor specificity, poor selectivity, and poor accuracy. Recently, a fluorometric assay based on aniline blue dye was developed and applied for the specific quantification of 1,3-β-glucans in mushrooms and foods [17,18,19]. This method has the advantages of high specificity, high selectivity, and convenience. However, the calibration range (0–10 µg/mL, curdlan) of the developed fluorometric assay is limited [17,18], which is not appropriate for the determination of a high content of 1,3-β-glucans in mushrooms. An extended calibration range is required for the quantification of 1,3-β-glucans in edible mushrooms. Additionally, the detection system of the developed fluorometric assay has seldom been optimized and fully validated. Therefore, in the present study, an improved fluorometric assay based on aniline blue dye for the accurate, specific, and rapid quantification of 1,3-β-glucans in edible mushrooms was developed and fully validated. Furthermore, the improved fluorometric assay was applied for the quantitative evaluation of water soluble 1,3-β-glucans extracted from *D*. *indusiata* by UAE with RSM. The results suggest that the improved fluorometric assay has great potential to be used as a routine method for the quantitative evaluation of 1,3-β-glucans in edible mushrooms and that ultrasound-assisted extraction is effective for their extraction.

## 2. Materials and Methods

### 2.1. Material and Chemicals

The fruiting bodies of *D. indusiata* were purchased from a local market in Ya’an, China. The sample was dried at 50 °C for 2 days and then milled into flour. Subsequently, the flour was passed through a 60-mesh screen and stored at −20 °C for further analysis.

Aniline blue, pectin (galacturonic acid ≥74.0%), chitosan, glucose, soluble starch, dextran (molecular weight, 64,000–76,000 Da), fucoidan, and bovine serum albumin (BSA) were purchased from Sigma-Aldrich (St. Louis, MO, USA). Carboxymethyl cellulose (CM-cellulose), β-glucan from barley (purity, ≥95%), arabinoxylan (purity, ~90%), inulin (purity, ≥95%), glucomannan (purity, ≥98%), pectic galactan, and curdlan (1,3-β-glucan; purity, >95%) were obtained from Megazyme (Wicklow, Ireland). All other reagents and chemicals used were of analytical grade.

### 2.2. Fluorometric Assay for 1,3-β-glucans Quantification

#### 2.2.1. Optimization, Calibration Curve, and Specificity of the Fluorometric Assay

The fluorometric assay for 1,3-β-glucans quantification was optimized and improved based on the previously reported methods [17,20]. Briefly, the reagent system of the fluorometric assay was composed of 2.5 mL dye solution (0.5% aniline blue in ultra-pure water, *w/v*), 3.5 mL glycine/NaOH buffer (1.0 M glycine and 1.25 M NaOH), 1.5 mL 2 M HCl, and 12.5 mL ultra-pure water. The mixture was then stored in the dark for 2 days to decolorize it from blue to yellow. For the quantification of 1,3-β-glucans, 300 µL of the sample solution was denatured with 30 µL of different concentrations of NaOH solution at 80 °C for different incubation times. Then, 100 µL of the sample was added into 900 µL of the fluorometric assay solution and incubated at 50 °C for 30 min to accelerate the 1,3-β-glucan-fluorochrome complex formation. Finally, after centrifugation at 4000× *g* for 15 min, 200 µL of supernatant was transferred to the black 96-well fluorescence plate and measured at an emission wavelength of 495 nm with an excitation wavelength of 405 nm by using a Varioskan Flash Multimode Reader with a scanning speed of 15 s (Thermo Fisher, Waltham, MA, USA). To optimize the detection conditions of the fluorometric assay, the effects of different concentrations (the final concentrations of 0.05 M, 0.3 M, and 0.6 M, respectively) of NaOH and different incubation times (30 min, 45 min, and 60 min, respectively) for the sample denaturation on the fluorescence intensity were investigated.

Furthermore, curdlan was used as the 1,3-β-glucan standard to build a calibration curve. The binding specificity of the fluorescent dye (aniline blue) to 1,3-β-glucans was examined by comparing the aniline-blue-induced fluorescence intensities among pectin, CM-cellulose, chitosan, glucose, soluble starch, barley β-glucan, dextran, arabinoxylan, inulin, glucomannan, pectic galactan, and fucoidan at the concentration of 300 µg/mL and 600 µg/mL, respectively. Additionally, the effect of simulative protein on the fluorometric assay was tested by adding 150 µL of BSA at 10%, 50%, 100%, 150%, 200%, 500%, and 1000% (*w/w*) of curdlan concentrations into 150 µL of 300 or 600 µg/mL curdlan solutions.

#### 2.2.2. Precision, Repeatability, Stability, Accuracy, Limit of Determination (LOD), and Limit of Quantification (LOQ) of the Improved Fluorometric Assay

The precision was determined via evaluating the variation of intra-day and inter-day measurements. The intra-day precision was determined via repeating the analysis of the curdlan standard solution six times within one day, and the inter-day precision was determined in duplicate on three successive days. The repeatability was confirmed with the preparation and analysis of six parallel curdlan standard solutions and fluorometric assay solutions. To determine the stability, the curdlan standard solution was stored at 4 °C for analysis on three successive days. Variations were expressed by the relative standard deviations (RSDs). Recovery was used to evaluate the accuracy. Known amounts of curdlan standard were added to a certain amount (1.0 g) of *D. indusiata* and then extracted with 0.05 M NaOH solution and analyzed in triplicate. Afterwards, the limit of determination (LOD) and limit of quantification (LOQ) were calculated from twenty blanks using Long and Winefordner formulas from the International Union of Pure and Applied Chemistry (IUPAC) definitions [21].

### 2.3. Ultrasound-Assisted Extraction of Water Soluble 1,3-β-glucans from D. indusiata

#### 2.3.1. Single Factor Experimental Design

The water soluble 1,3-β-glucans were extracted from *D. indusiata* by ultrasound-assisted extraction with an Ultrasonic Processor (650 W, 24 kHz, Scientz company, NingBo, China). Briefly, 1.0 g of *D. indusiata* powder was extracted with distilled water by UAE, and the effects of the ultrasound amplitude (10%, 25%, 40%, 55%, and 70%), ultrasound time (2, 5, 10, 15, and 20 min), and ratio of liquid to raw material (10, 20, 30, 40, and 50 mL/g) on the yield of water soluble 1,3-β-glucans were investigated using a single factor experimental design. After the extraction, solutions were centrifuged at 4000× *g* for 15 min. The supernatant was collected and precipitated by 4 volumes of 95% ethanol overnight. Then the polysaccharides were re-dissolved in 100 mL of distilled water and stored at 4 °C for further analysis. The amount of water soluble 1,3-β-glucans was measured by the improved fluorometric assay, and the yield of the water soluble 1,3-β-glucans was calculated using the following equation:

(1) $$\mathrm{Extraction}\;\mathrm{yield}\;\left(\%\right)=\frac{W_{1}}{W_{0}}\;\times 100$$

where *W*_1_ was the amount of water soluble 1,3-β-glucans in *D. indusiata* (mg) and *W*_0_ was the dry weight (mg) of the *D. indusiata* sample.

#### 2.3.2. Box–Behnken Experimental Design

Based on the results of the single factor experiments, a three-level Box–Behnken experimental design (BBD) with three factors was applied to optimize the UAE conditions. The ultrasound amplitude (*X*_1_, %), ultrasound time (*X*_2_, min), and ratio of liquid to raw material (*X*_3_, mL/g) were preferred for the independent variables. According to the BBD, 17 experimental runs, with 1 block and 5 center points, were performed. The variables and their levels, with both coded and actual values, are presented in Table 1. The obtained data were analyzed using the statistical analysis of the Design Expert 8.0.5 software (Stat-Ease Inc., Minneapolis, MN, USA). The significance of the model was evaluated by analysis of variance (ANOVA). The experimental data from the BBD are explained by the second-order polynomial model as follows [12]:

(2) $$Y=\;A_{0}\;+\;\sum_{i=1}^{3}A_{i}X_{i}\;+\;\sum_{i=1}^{3}A_{ii}X^{2}{}_{i}\;+\;\sum_{i=1}^{2}\sum_{j=i+1}^{3}A_{ij}X_{i}X_{j}$$

where Y is the predicted response; *X_i_* and *X_j_* are different variables (*i* ≠ *j*); and *A*_0_, *A_i_*, *A_ii_*, and *A_ij_* are the regression coefficients for the intercept, linearity, square, and interaction, respectively.

#### 2.3.3. Hot Water Extraction of Water Soluble 1,3-β-glucans from *D. indusiata*

Hot water extraction of water soluble 1,3-β-glucans from *D. indusiata* was performed according to a previously optimized method with slight modifications [9]. Briefly, 1.0 g of *D. indusiata* powder was extracted with 35 mL water at 95 °C for 2.5 h. After centrifugation at 4000× *g* for 15 min, the supernatant was collected and precipitated by 4 volumes of 95% ethanol overnight. The polysaccharides were re-dissolved in pure water and stored at 4 °C for further analysis. The amount of water soluble 1,3-β-glucans and total polysaccharides were measured by the improved fluorometric assay and the phenol sulfuric acid method, respectively.

### 2.4. Statistical Analysis

All the experiments were conducted in triplicate. Statistical analysis was performed using the Design Expert 8.0.5 software (Stat-Ease Inc., Minneapolis, MN, USA). The significance of the model was evaluated by analysis of variance (ANOVA). Values of *p* < 0.05 were considered as statistically significant.

## 3. Results and Discussions

### 3.1. Optimal Conditions, Calibration Curve, and Specificity of the Fluorometric Assay

**Figure 1:**
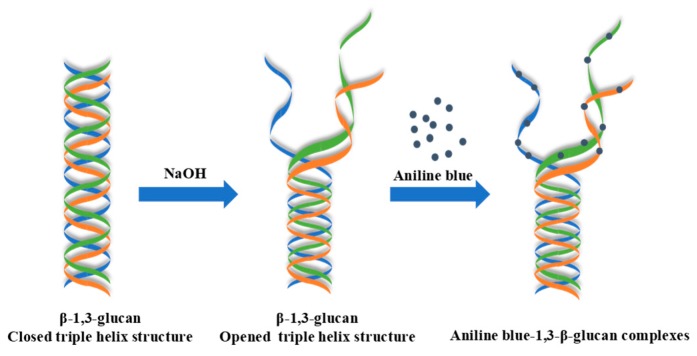
Basic chemical principles of the fluorometric assay for the determination of 1,3-β-glucans.

The basic chemical principles of the fluorometric assay for the determination of 1,3-β-glucans is shown in Figure 1. Generally, the chain conformation of 1,3-β-glucan exhibits as a triple-helix structure in aqueous solution. The triple-helix 1,3-β-glucan can be changed into an opened triple helix rather than a single helix by treating it with NaOH. The 1,3-β-glucan with a single-helix conformation (opened triple-helix structure) preferentially and specifically reacts with aniline blue in solution and is eventually labeled, whereas the unlabeled dye is decolorized. In addition, the aniline blue fluoresces only when it binds to 1,3-β-glucan in solution [17]. The fluorescence intensity of aniline blue–1,3-β-glucan complexes can be affected by several factors of the solution, such as ionic strength, pH, and temperature [22,23]. Therefore, we maintained the similar proportion of the compositions in the aniline blue mixture, as described previously [20]. Additionally, the concentration of NaOH and the incubation time for 1,3-β-glucan denaturation also significantly affect the formation of aniline blue–1,3-β-glucan complexes [22,24]. Therefore, the effects of different concentrations of NaOH and incubation times for the sample denaturation on the fluorescence intensity were investigated. The results showed that the fluorescence intensities of curdlan (300 μg/mL) and *D. indusiata* 1,3-β-glucans treated with 0.05 M, 0.3 M, and 0.6 M NaOH for 30 min were 55.2 ± 2.1 (n = 3), 157.9 ± 2.5 (n = 3), and 157.7 ± 1.7 (n = 3) and 11.3 ± 0.4 (n = 3), 57.1 ± 0.4 (n = 3), and 59.1 ± 0.6 (n = 3), respectively. In accordance with previous reports, these results suggest that 0.3 M NaOH is applicable for the denaturation of 1,3-β-glucans [24]. The denaturation of 1,3-β-glucans is necessary for their quantification by the improved fluorometric assay based on aniline blue dye, which is different from that of previous studies [20]. In addition, the fluorescence intensities of curdlan (300 μg/mL) and *D. indusiata* 1,3-β-glucans treated with 0.6 M NaOH for 30 min, 45 min, and 60 min were 159.9 ± 3.1 (n = 3), 157.7 ± 1.6 (n = 3), and 144.3 ± 4.1 (n = 3) and 59.8 ± 2.2 (n = 3), 58.6 ± 0.8 (n = 3), and 46.6 ± 1.8 (n = 3), respectively. These results suggest that the fluorescence intensities of the aniline blue–1,3-β-glucan complexes decreased with the increasing incubation time, which might be attributed to the degradation of 1,3-β-glucans under a high alkaline condition for a longer time [25,26]. Therefore, the optimal concentration of NaOH and the optimal incubation time for the sample denaturation were 0.6 M and 30 min, respectively.

**Figure 2:**
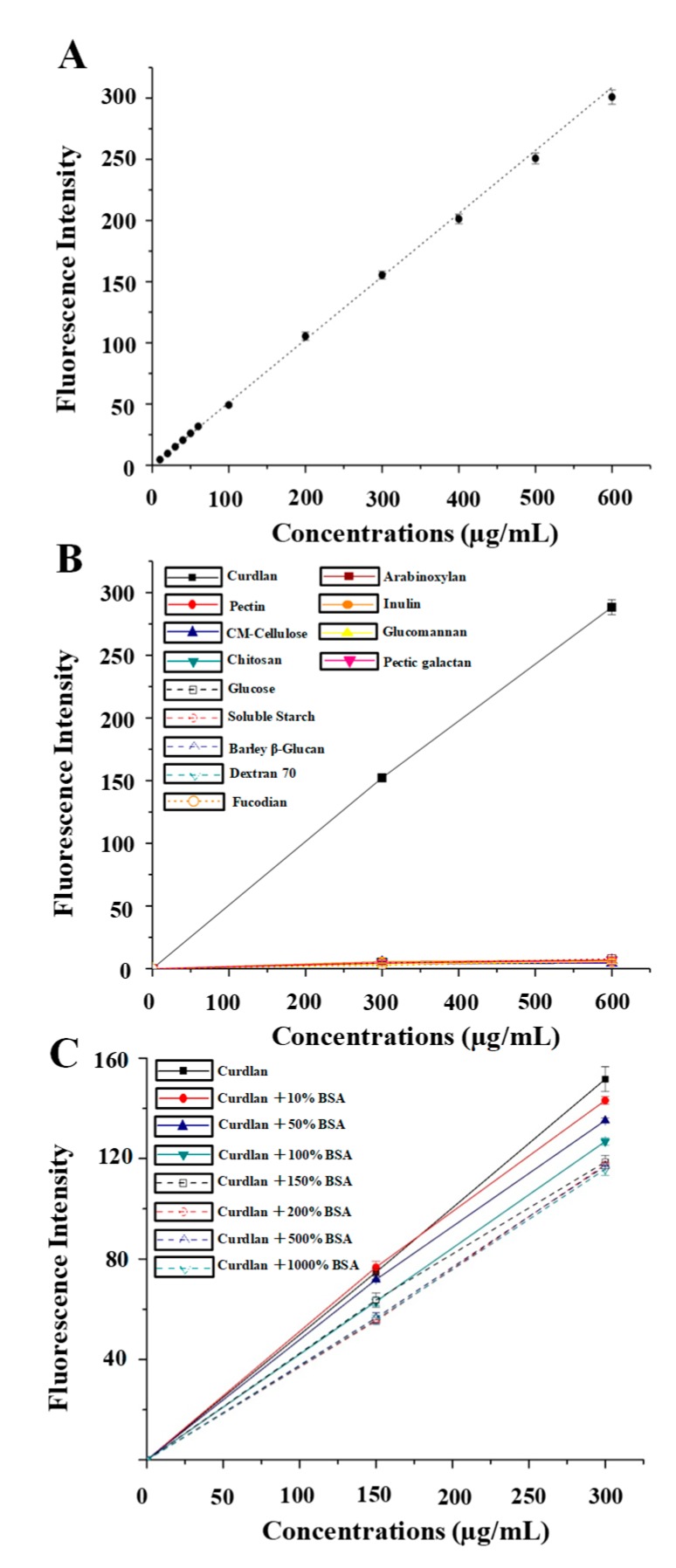
Calibration curve (**A**), specificity (**B**), and influence of protein on accuracy (**C**) of the improved fluorometric assay. All experiments were conducted in triplicate, and data are expressed in means ± standard deviations. The error bars are standard deviations.

To quantify the water soluble 1,3-β-glucans in *D. indusiata*, the curdlan standard was used to construct a calibration curve. Figure 2A shows a linear correlation of the fluorescence intensity of the curdlan concentration in the range of 10–600 µg/mL with *R*^2^ > 99.9%. Indeed, a linear correlation is also shown with *R*^2^ > 99.9% in the reduced range of 10–60 µg/mL. Compared to previous studies, the improved fluorometric assay showed a wider calibration range and superior linearity [17,18,19]. Some possible reasons for the improvement in the calibration range have been discussed [20]. Moreover, the specificity of the fluorescence binding of aniline blue with 1,3-β-glucans in the current working solution was demonstrated by comparing various carbohydrate polymers. As shown in Figure 2B, only the curdlan standard exhibited significant correlation signals, whereas pectin, CM-cellulose, chitosan, soluble starch, barley β-glucan, dextran, glucose, arabinoxylan, inulin, glucomannan, pectic galactan, and fucoidan showed almost background signals. Generally, only glucans containing the commonly specific 1,3-β-glucan structure exerted similar fluorescence profiles [17,20]. Additionally, mushrooms always contain proteins, which may interfere with fluorescence. Therefore, in order to examine the interference of protein from mushrooms, BSA was used as the model substance to test the influence of protein on the aniline blue fluorescence. As shown in Figure 2C, the fluorescence intensities of curdlan (the final concentrations of 150 µg/mL and 300 µg/mL) decreased 2.63% and 3.92% with 10% of BSA and 5.52% and 11.11% with 50% of BSA, respectively. This suggested that the interference of 50% BSA could be ignored at the lower concentration of curdlan (the final concentration of 150 µg/mL). However, the fluorescence intensity of curdlan was significantly affected with the increased BSA concentrations (Figure 2C). Therefore, the results indicated that protein was an interfering factor in the fluorometric assay, however, 10–50% of BSA in 150 µg/mL of curdlan had no significant effect on the accuracy, which is in good agreement with the previous report [17].

### 3.2. Method Validation of the Improved Fluorometric Assay

The improved fluorometric assay was fully validated in the present study. The LOD and LOQ of the improved fluorometric assay were calculated as 1.91 µg/mL and 6.29 µg/mL, respectively. The RSDs of the intra-day and inter-day precision and repeatability were 0.75% (n = 6), 1.67% (n = 6), and 3.14% (n = 6), respectively. The data suggested that the improved fluorometric assay had good precision and repeatability. Meanwhile, the RSD of the sample stability was 2.35% (n = 6), which indicated that the investigated curdlan standard solution was stable at 4 °C during the tested period. Further, the improved fluorometric assay had a good accuracy with a recovery of 98.7% (RSD, 3.3%, n = 3). Therefore, the improved fluorometric assay could be further applied for the accurate quantification of water soluble 1,3-β-glucans in *D. indusiata*.

### 3.3. Single Factor Experimental Analysis

**Figure 3:**
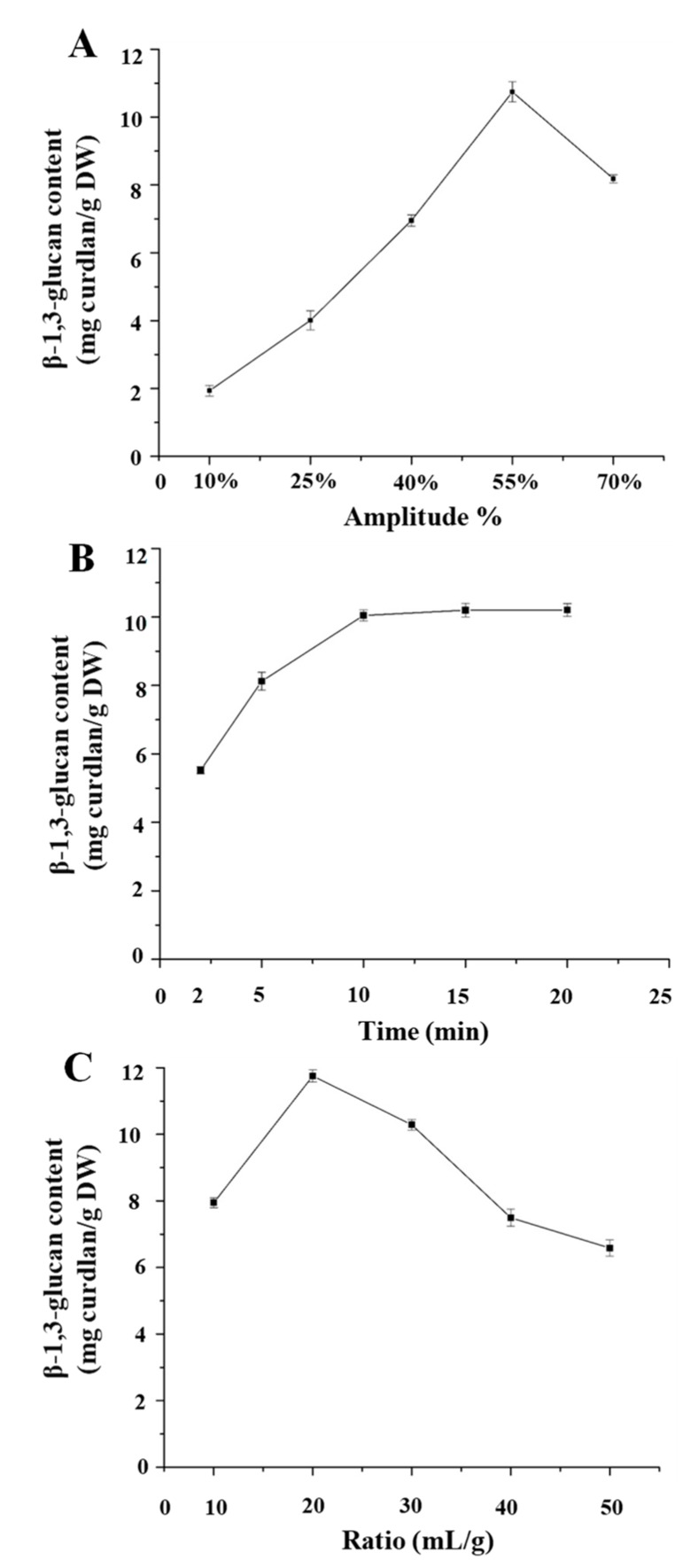
Effects of (**A**) ultrasound amplitude, (**B**) ultrasound extraction time, and (**C**) ratio of water to raw material on the extraction yield of water soluble 1,3-β-glucans. All experiments were conducted in triplicate, and data are expressed in means ± standard deviations. The error bars are standard deviations.

Generally, the factors of ultrasound amplitude, ultrasound extraction time, and the ratio of liquid to raw material significantly affect the yield of polysaccharides by UAE [12,14]. The effect of ultrasound amplitude on the yield of water soluble 1,3-β-glucans is shown in Figure 3A. Various ultrasound amplitudes (10, 25, 40, 55, and 70%) were examined, while the other extraction variables were kept as follows: ultrasound extraction time (10 min) and ratio of liquid to raw material (30 mL/g). The results showed that the yield of water soluble 1,3-β-glucans increased from 0.19% to 1.07% as the ultrasound amplitude increased from 10% to 55%, while the highest yield (1.07%) was achieved at the amplitude of 55%. Further, the yield decreased with the increasing ultrasound amplitude to 70%. Therefore, the ultrasound amplitude of 55% was selected as the optimal ultrasound amplitude. Additionally, the effect of the ultrasound extraction time (2, 5, 10, 15, and 20 min) on the yield of water soluble 1,3-β-glucans was studied with an ultrasound amplitude of 55% and a ratio of liquid to raw material of 30 mL/g. As shown in Figure 3B, the yield of water soluble 1,3-β-glucans increased from 0.55% to 1.04% as the extraction time increased from 2 to 20 min, while the highest yield (1.04%) was achieved in 10 min. There was no significant difference among 10, 15, and 20 min on the yield of water soluble 1,3-β-glucans. Therefore, in order to avoid energy consumption and reduce the extraction time, 10 min was selected as the optimal extraction time. Moreover, the effect of the ratios of liquid to raw material (10, 20, 30, 40, and 50 mL/g) on the yield of water soluble 1,3-β-glucans was studied with an ultrasound amplitude of 55% and an ultrasound extraction time of 10 min. As shown in Figure 3C, the yield of water soluble 1,3-β-glucans increased from 0.79% to 1.18% as the ratios of liquid to raw material increased from 10 to 20 mL/g, while the highest yield (1.18%) was achieved at the ratio of 20 mL/g. Furthermore, the yield decreased with the increasing ratio of liquid to raw material to 50 mL/g. Therefore, the ratio of 20 mL/g was selected as the optimal ratio of liquid to raw material.

### 3.4. Model Fitting and Statistical Analysis

According to the results of single factor experiments, BBD with seventeen runs was applied to optimize the three independent extraction variables, namely ultrasound amplitude (*X*_1_), ultrasound extraction time (*X*_2_), and ratio of liquid to raw material (*X*_3_). Table 1 shows the BBD matrix and the experimental data. Using multiple regression analysis, Design Expert 8.0.5 generated a second-order polynomial equation to express the relationship between the process and the response. The final equation in terms of coded factors was as follows:

(3) $$Y=11.63+\;0.50X_{1}+0.94X_{2}++0.33X_{3}-0.11\;X_{1}X_{2}-0.068X_{1}X_{3}+0.47X_{2}X_{3}\;-1.79X_{1}^{2}-0.48X_{2}^{2}-1.76X_{3}^{2}$$

where Y represents the yield of water soluble 1,3-β-glucans in *D. indusiata* and *X*_1_, *X*_2_, and *X*_3_ are the ultrasound amplitude, the ultrasound extraction time, and the ratio of liquid to raw material, respectively.

The statistical significance of the second-order polynomial equation was analyzed by using a one-way ANOVA. In the BBD analysis, the *F*-value and *p*-value were used to check the significance of each coefficient. As shown in Table 2, the quadratic regression model has a high *F*-value (40.75) and a very low *p*-value (*p* < 0.0001), which indicates that the fitness of the model is highly significant [27]. The lack of fit *F*-value of 3.07 and *P*-value of 0.1534 (*p* > 0.05) implies that the lack of fit was not significant. Therefore, the results indicate that the model equation is adequate for predicting the yield of water soluble 1,3-β-glucans from *D. indusiata* under the conditions of any combination of the variable values [11]. In addition, the low value of the coefficient variation (*C.V.*, 3.58%) and the high value of adequate (adeq.) precision (16.631) indicates that this model has good precision and reliability [12,28]. The coefficient of determination (R^2^) and adjusted coefficient of determination (R^2^*_adj_*) were 0.9795 and 0.9532, respectively, which are close to 1.0, indicating that this polynomial model has adequate accuracy and general applicability [29]. Moreover, the linear coefficients (*X*_1_, *X*_2_, and *X*_3_), interaction coefficient (*X*_2_*X*_3_), and quadratic term coefficients (*X*_12_, *X*_22_, and *X*_32_) were significant (*p* < 0.05), while the interaction coefficients (*X*_1_*X*_2_ and *X*_1_*X*_3_) had no significant influence (*p* > 0.05) on the extraction.

### 3.5. Analysis of Response Surface Plot and Contour Plot

**Figure 4:**
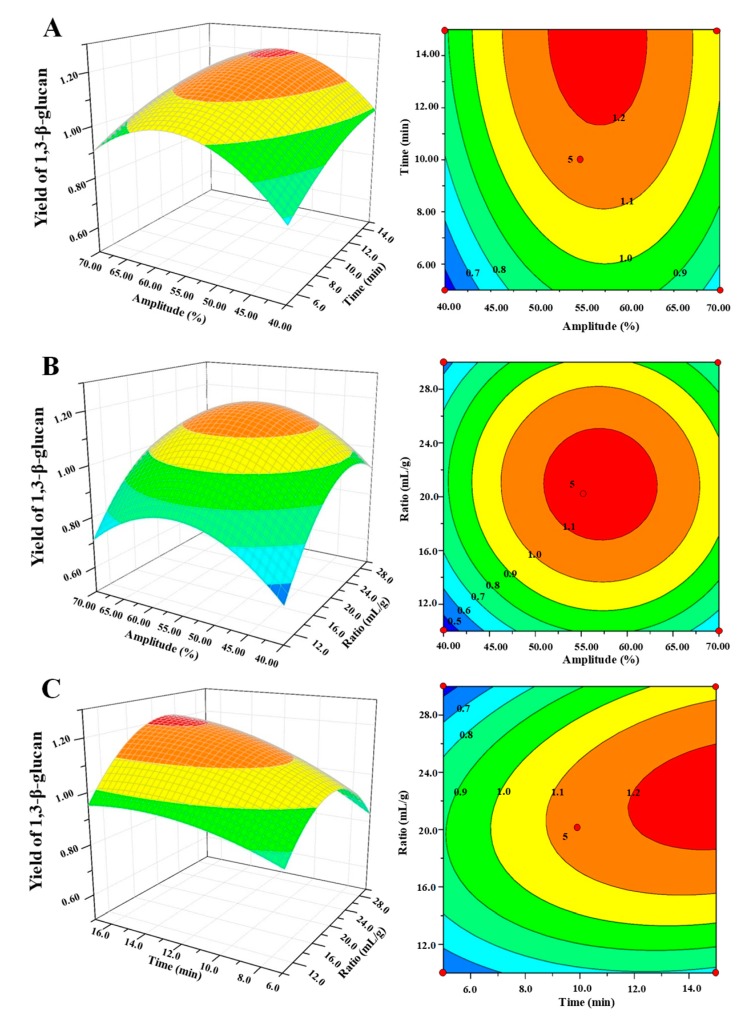
Three-dimensional response surface (left) and two-dimensional contour (right) plots showing the interaction effects on the extraction yield of water soluble 1,3-β-glucans: (**A**) ultrasound amplitude and ultrasound extraction time; (**B**) ultrasound amplitude and ratio of water to raw material; and (**C**) ultrasound extraction time and ratio of water to raw material.

The mutual interactions of independent and dependent variables on the yield of water soluble 1,3-β-glucans from *D. indusiata* could be visualized by three-dimensional (3D) response surface plots and two-dimensional contour plots (Figure 4). Generally, the shapes of the contour plots indicate whether the mutual interactions between variables are significant or not. Circular contour plots indicated that the interactions were non-significant, whereas elliptical contours demonstrated the interactions were significant [30]. The effect of the ultrasound amplitude and ultrasound extraction time on the yield of water soluble 1,3-β-glucans is shown in Figure 4A. The yield increased as the ultrasound amplitude increased from 40% to 56% and then decreased as the amplitude constantly increased to 70%. In addition, the yield increased as the extraction time increased from 5 min to 10 min and then increased slightly from 10 min to 15 min. Further, the effect of the ultrasound amplitude and ratio of liquid to raw material on the yield of water soluble 1,3-β-glucans is shown in Figure 4B. A circular contour plot indicated that interactions between the ultrasound amplitude and ratio of liquid to raw material were not significant, which is in accordance with the results in Table 2. Meanwhile, Figure 4C shows the effect of the extraction time and ratio of liquid to raw material on the yield of water soluble 1,3-β-glucans. The obvious elliptical contour plot and *P*-value of 0.03 (Table 2) indicate that the interactions between the extraction time and ratio of liquid to raw material were significant.

### 3.6. Verification of the Predictive Model and Comparison of UAE and HWE

The optimal values (ultrasound amplitude of 55.57%, ultrasound extraction time of 15.0 min, and ratio of liquid to raw material of 22.27 mL/g) of the variables were obtained using the quadratic polynomial model. Considering the operability in the actual processing procedure, the confirmatory experiment was performed under the following conditions: ultrasound amplitude of 56.0%, ultrasound extraction time of 15.0 min, and ratio of liquid to raw material of 22.0 mL/g. Under these optimal UAE conditions, the yield of water soluble 1,3-β-glucans was 1.20% (RSD = 0.54%, n = 3) and the yield of total polysaccharides was 5.41% (RSD = 1.14%, n = 3). The good correlation between the experimental (1.20%) and predicted (1.22%) values confirmed that the response model accurately and adequately represented the yield of water soluble 1,3-β-glucans. Furthermore, in order to evaluate the extraction efficiency of the developed UAE method, the HWE was performed and compared. The yields of water soluble 1,3-β-glucans and total polysaccharides from *D. indusiata* were 1.03% (RSD = 0.94%, n = 3) and 3.12% (RSD = 1.22%, n = 3), respectively, which were significantly lower than those of the UAE method. The results suggest that UAE was more effective for the extraction of water soluble 1,3-β-glucans and total polysaccharides from *D. indusiata* than HWE. Additionally, the yield of total polysaccharides from *D. indusiata* in the present study was significantly lower than that of previously reported results under the similar HWE conditions [9], which might be attributed to the different resources of *D. indusiata* used.

## 4. Conclusions

In this study, an improved fluorometric assay based on aniline blue dye for accurate and rapid quantification of 1,3-β-glucans in edible mushrooms was developed and fully validated. Furthermore, the improved fluorometric assay was successfully applied for the quantitative evaluation of water soluble 1,3-β-glucans extracted from *D. indusiata* by ultrasound-assisted extraction and hot water extraction. The results indicate that ultrasound-assisted extraction is effective for extracting 1,3-β-glucans from edible mushrooms. Furthermore, the results suggest that the improved fluorometric assay has great potential to be used as a routine method for the quantification of 1,3-β-glucans in edible mushrooms, which is helpful for quality evaluations of 1,3-β-glucans in mushrooms and their related products.

## Figures and Tables

**Table 1:** Box–Behnken design with independent variables and observed and predicted values.

Runs	Levels of Independent Factors ^a^			Extraction Yield %	
	*X*_1_ (%)	*X*_2_ (min)	*X*_3_ (mL/g)	Observed ^b^	Predicted
1	1 (70.00)	1 (15.00)	0 (20.00)	1.055	1.069
2	0 (55.00)	0 (10.00)	0 (20.00)	1.138	1.163
3	0 (55.00)	0 (10.00)	0 (20.00)	1.158	1.163
4	0 (55.00)	0 (10.00)	0 (20.00)	1.178	1.163
5	−1 (40.00)	0 (10.00)	1 (30.00)	0.762	0.799
6	0 (55.00)	1 (15.00)	−1 (10.00)	0.929	0.952
7	−1 (40.00)	−1 (5.00)	0 (20.00)	0.795	0.781
8	0 (55.00)	0 (10.00)	0 (20.00)	1.199	1.163
9	0 (55.00)	1 (15.00)	1 (30.00)	1.138	1.114
10	1 (70.00)	0 (10.00)	−1 (10.00)	0.869	0.832
11	1 (70.00)	0 (10.00)	1 (30.00)	0.875	0.886
12	0 (55.00)	−1 (5.00)	1 (30.00)	0.855	0.832
13	0 (55.00)	0 (10.00)	0 (20.00)	1.142	1.163
14	1 (70.00)	−1 (5.00)	0 (20.00)	0.892	0.905
15	0 (55.00)	−1 (5.00)	−1 (10.00)	0.835	0.860
16	−1 (40.00)	1 (15.00)	0 (20.00)	1.003	0.991
17	−1(40.00)	0 (10.00)	−1 (10.00)	0.875	0.886

^a^*X*_1_, ultrasound amplitude (%); *X*_2_, ultrasound extraction time (min); *X*_3_, ratio of water to raw material (mL/g); ^b^ mean of triplicate determination.

**Table 2:** Analysis of variance of regression equation and coefficients.

Source ^a^	Sum of Squares	d*f ^b^*	Mean Square	*F-*Value	*p*-Value ^c^
Model	40.75	9	4.53	37.20	<0.0001^**^
*X*_1_	2.03	1	2.03	16.68	0.0047^**^
*X*_2_	7.00	1	7.00	57.52	0.0001^**^
*X*_3_	0.89	1	0.89	7.35	0.0302^*^
*X*_1_ *X*_2_	0.051	1	0.051	0.42	0.5385
*X*_1_ *X*_3_	0.018	1	0.018	0.15	0.7087
*X*_2_ *X*_3_	0.90	1	0.90	7.37	0.0300^*^
*X*_1_^2^	13.43	1	13.43	110.38	<0.0001^**^
*X*_2_^2^	0.97	1	0.97	8.01	0.0254^*^
*X*_3_^2^	13.01	1	13.01	106.88	<0.0001^**^
Residual error	0.85	7	0.12		
Lack of fit	0.59	3	0.20	3.07	0.1534
Pure error	0.26	4	0.064		
Correlation total	41.60	16			

*R*^2^ = 0.9795; *R*^2^*_adj_* = 0.9532; coefficient of variation = 3.58%; adeq. precision = 16.631. ^a^ *X*_1_, ultrasound amplitude (%); *X*_2_, ultrasound extraction time (min); *X*_3_, ratio of water to raw material (mL/g); d*f* *^b^*, the degree of freedom. ^c^ *significant difference (*p* < 0.05); **extremely significant difference (*p* < 0.01).
